# SIRT1 Interacts with Prepro-Orexin in the Hypothalamus in SOD1G93A Mice

**DOI:** 10.3390/brainsci12040490

**Published:** 2022-04-11

**Authors:** Gan Zhang, Rong Liu, Zhaofu Sheng, Yonghe Zhang, Dongsheng Fan

**Affiliations:** 1 Department of Neurology, Peking University Third Hospital, Beijing 100191, China; g.zhang@bjmu.edu.cn (G.Z.); liurong200800@163.com (R.L.); 2 Beijing Municipal Key Laboratory of Biomarker and Translational Research in Neurodegenerative Disease, Beijing 100191, China; jianbingxuan@163.com; 3 Department of Pharmacology, School of Basic Medical Science, Peking University, Beijing 100191, China

**Keywords:** amyotrophic lateral sclerosis, SIRT1, prepro-orexin, SOD1G93A mice

## Abstract

The participation of silent mating type information regulation 2 homolog 1 (SIRT1) in amyotrophic lateral sclerosis (ALS) has been reported in many studies. However, the role of the expression and function of SIRT1 in the hypothalamus in ALS remains unknown. In the current study, we performed western blot, co-immunoprecipitation and immunofluorescence analyses to determine the expression and in-depth mechanism of SIRT1 in the hypothalamus in SOD1G93A transgenic mice. We found that SIRT1 was overexpressed in the hypothalamus after motor symptom onset. In addition, SIRT1 interacted with prepro-orexin, a molecule involved in energy balance and the sleep/wake cycle, in both preclinical and clinical ALS regardless of whether SIRT1 levels were elevated. These findings indicate that SIRT1 might participate in sleep and metabolic changes in ALS, suggesting that SIRT1 is a new target for ALS treatment.

## 1. Introduction

Amyotrophic lateral sclerosis (ALS) is a neurodegenerative disease characterized by progressive loss of motor neurons and muscle atrophy leading to paralysis and death [1]. Recent evidence has indicated that ALS involves more than just the motor system; lesions have also been found in nonmotor areas of the central nervous system [2,3,4]. One molecule involved in the pathological changes of ALS is silent mating type information regulation 2 homolog 1 (SIRT1), a member of the sirtuin family of NAD-dependent protein deacetylases that adjusts transcriptional networks associated with a wide range of aging-related biological functions [5]. SIRT1 has been suggested to be upregulated in many brain areas in ALS model [6]. However, little evidence is available regarding the expression and function of SIRT1 in the hypothalamus, an area closely associated with multiple nonmotor symptoms, or regarding the role of SIRT1 in the onset and progression of ALS [7,8]. According to our previous study, the levels of orexins, hypothalamus-derived neuropeptides that are well known to regulate energy balance and the sleep/wake cycle (which is regulated by SIRT1), are increased in the hypothalamus in SOD1G93A mice [9]. Therefore, we hypothesized that a change in SIRT1 expression in the hypothalamus might be connected with orexin dysregulation.

To test our hypothesis, we used SOD1G93A transgenic mice, applied western blotting to detect SIRT1 expression in the hypothalamus and used immunofluorescence and co-immunoprecipitation to detect whether SIRT1 and prepro-orexin interact.

## 2. Materials and Methods

### 2.1. Experimental Animals

The transgenic SOD1G93A mice used in this study were bred from male hemizygous SOD1G93A mice (B6SJL-Tg (SOD1-G93A) 1 Gur/J) and female B6SJL/F1 hybrids purchased from Jackson Laboratories (Bar Harbor, ME, USA). The SOD1G93A mice were genotyped as previously reported [10]. Nontransgenic littermates served as controls. Tests were performed at two time points: 90 days after birth (before disease onset), which was defined as the presymptomatic stage, and 120 days after birth, which was defined as the symptomatic stage for SOD1G93A transgenic mice according to our previous study [9]. There were 4–6 animals in each group at each time point. Our mice were housed in a controlled environment at 22 ± 1 °C with a 12 h:12 h light:dark cycle. All mice were deeply anesthetized with 7% chloral hydrate in saline (350 mg/kg intraperitoneally) when sacrificed. All animal procedures were conducted in accordance with the guidelines of the Institutional Animal Care and Use Committee.

### 2.2. Western Blotting

Proteins were extracted from the hypothalamus (*n* = 4–6/group) and then processed and quantified with a Total Protein Extraction Kit (Applygen, Beijing, China) and a Bicinchoninic Acid Protein Assay Kit (Applygen). Equal amounts of protein samples (20 µg) were separated by SDS-PAGE and transferred to a polyvinylidene fluoride membrane (Millipore, Burlington, MA, USA). The membrane was blocked with 3% bovine serum albumin (BSA) dissolved in Tris-buffered saline (TBS) containing 0.1% Tween 20 for approximately 1 h. Then, the processed membrane was incubated with anti-orexin-prepro (1:500, Millipore), anti-SIRT1 (1:5000, Abcam, Cambridge, UK), or anti-β-actin (1:5000, Earthox, Millbrae, CA, USA) antibodies in a 4 °C environment overnight. The membrane was incubated with goat anti-rabbit or anti-mouse secondary antibodies (1:10,000, LI-COR, Lincoln, NE, USA) at 37 °C for one hour. The immunoreactive bands were quantified with an Odyssey infrared imaging system (LI-COR).

### 2.3. Immunofluorescence

After anesthetization and perfusion, the brain tissues of mice were postfixed in paraformaldehyde. Then, the lateral hypothalamus was located using a mouse brain atlas [11], and 8-µm-thick frozen coronal sections were cut through this region (*n* = 4–6/group). For prepro-orexin and SIRT1 double staining, the sections were incubated with rabbit anti-orexin-prepro (1:250, Millipore) and mouse anti-SIRT1 (1:1000, Abcam) at 4 °C for 24 h. Then, the sections were incubated with secondary Alexa 594/488-labeled goat anti-rabbit/mouse IgG antibodies (1:500, Jackson ImmunoResearch, West Grove, PA, USA) for 1 h. The nuclei were counterstained with DAPI solution (1 mg/mL, Vector Laboratories, Burlingame, CA, USA) for 10 min. The sections were mounted and examined with a laser scanning confocal microscope (Leica, Wetzlar, Germany).

### 2.4. Co-Immunoprecipitation

Briefly, proteins were extracted (*n* = 4–6/group), adjusted for concentration and then precleared by incubation with normal mouse IgG and protein A/G agarose beads. After centrifugation, the supernatant was incubated with protein A/G agarose beads and anti-orexin-prepro (Millipore) or anti-SIRT1 (Abcam) antibodies overnight at 4 °C. The immunocomplexes were washed with RIPA buffer, eluted by boiling in SDS sample buffer, and then subjected to western blotting.

### 2.5. Statistical Analysis

All data are expressed as the means ± standard errors. Independent-sample *t*-tests were performed by SPSS 19.0. *p* < 0.05 was considered to indicate significance.

## 3. Results

### 3.1. SIRT1 Levels Are Increased in the Hypothalami of Symptomatic SOD1G93A Transgenic Mice

**Figure 1:**
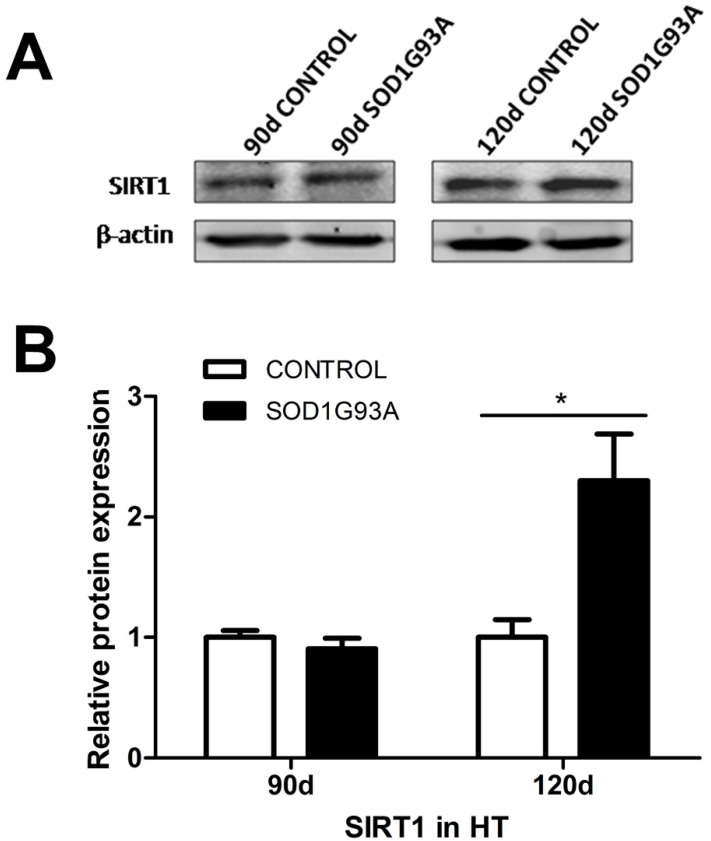
SIRT1 levels are increased in symptomatic SOD1G93A transgenic mice. (**A**) Protein expression levels of SIRT1 in the hypothalamus between SOD1G93A mice and control mice at 90 days and 120 days, as evaluated by western blotting. (**B**) Statistical results of SIRT1 expression between SOD1G93A mice and control mice at 90 days and 120 days. The data are expressed as the mean ± standard error (*n* = 4–6/group). * *p* < 0.05. HT, hypothalamus.

There were markedly higher SIRT1 levels in the hypothalami of SOD1G93A transgenic mice at the symptomatic stage (120 days after birth) than in controls (2.30 ± 0.39 vs. 1.00 ± 0.15, *p* < 0.05). However, there was no significant difference between SOD1G93A transgenic mice at the presymptomatic stage (90 days after birth) and control mice of the same age. The hypothalamic SIRT1 protein expression levels in SOD1G93A mice and control mice are shown in Figure 1A, and the statistical analysis results are shown in Figure 1B.

### 3.2. Prepro-Orexin Interacts with SIRT1 in the Hypothalamus in SOD1G93A Transgenic Mice

**Figure 2:**
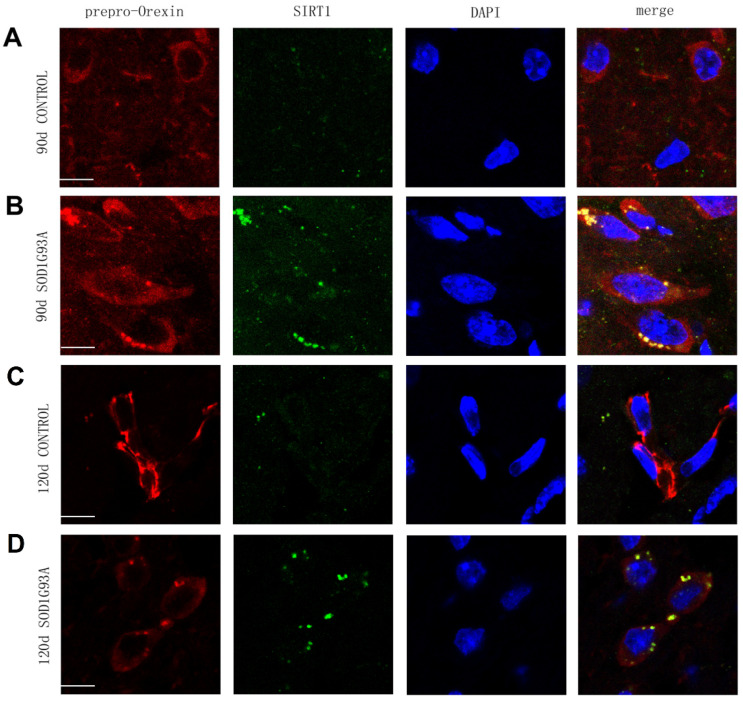
Immunofluorescence demonstrating that prepro-orexin interacts with SIRT1 in the cytoplasm of neurons in lateral hypothalamus in SOD1G93A transgenic mice. The merged image shows the colocalization (yellow) of prepro-orexin and SIRT1 in the hypothalamus in 90 days (**B**) and 120 days SOD1G93A transgenic mice (**D**) but not in 90-d (**A**) and 120-d control mice (**C**). Scale bar = 10 μm. Prepro-orexin (red); SIRT1 (green); DAPI (blue).

**Figure 3:**
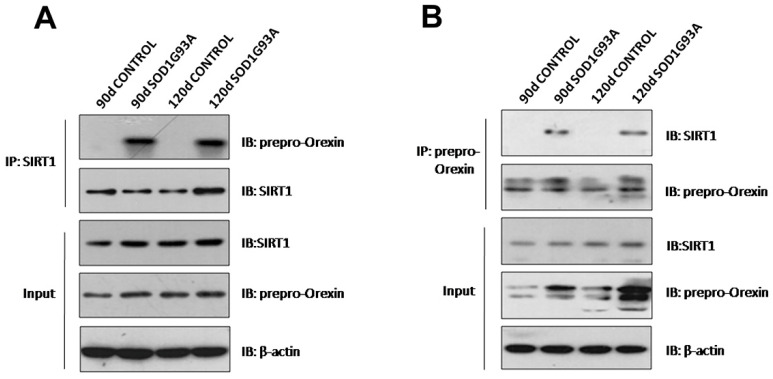
Co-immunoprecipitation confirmed that prepro-orexin interacts with SIRT1 in the hypothalamus in SOD1G93A transgenic mice. (**A**) Immunoprecipitation was performed using anti-SIRT1 antibodies. (**B**) Immunoprecipitation was performed using an anti-prepro-orexin antibody.

To examine whether there was an interaction between prepro-orexin and SIRT1 in the hypothalamus, we performed double immunostaining and co-immunoprecipitation. Double immunostaining showed colocalization of prepro-orexin and SIRT1 in the cytoplasm of neurons in the lateral hypothalamus in both presymptomatic (90 days after birth) and symptomatic (120 days after birth) SOD1G93A transgenic mice (Figure 2B,D) but not in controls (Figure 2A,C). In co-immunoprecipitation, the results obtained by immunoprecipitation using anti-SIRT1 (Figure 3A) and those obtained by immunoprecipitation using anti-orexin-prepro (Figure 3B) identified an interaction between these two proteins in the hypothalami of SOD1G93A transgenic mice at the two stages. However, the same phenomenon did not appear in the littermate controls.

## 4. Discussion

In this study, we tested whether SIRT1 levels are increased in the hypothalamus and whether SIRT1 interacts with orexin, which is dysregulated in the hypothalamus, in SOD1G93A mice. Although we observed increased SIRT1 levels only in symptomatic SOD1G93A mice, we demonstrated that prepro-orexin colocalized and interacted with SIRT1 in the hypothalamus of SOD1G93A mice at both the symptomatic stage and presymptomatic stage regardless of whether SIRT1 expression was increased.

SIRT1 participates in a wide range of physiological activities in the nervous system in many conditions. Previous evidence has revealed that SIRT1 levels in the cortex, hippocampus, thalamus and spinal cord are increased in SOD1G93A mice, suggesting that SIRT1 is involved in pathological changes in the central nervous system in ALS [6]. However, there is still a lack of evidence regarding the expression of SIRT1 in the hypothalamus. Hypothalamic SIRT1 plays important roles in metabolism and sleep/wake regulation under physiological conditions. In addition, recent findings suggested the importance of the hypothalamus in ALS. For example, Gorges et al. found that hypothalamic lesions are related to the onset age of ALS [8]. Therefore, our findings of increased SIRT1 levels in the hypothalamus not only supplement the findings of previous research that SIRT1 levels are increased in other areas in the brain [6], but also suggest the important influence of SIRT1 on ALS in the hypothalamus, the crucial structure for this disease.

**Figure 4:**
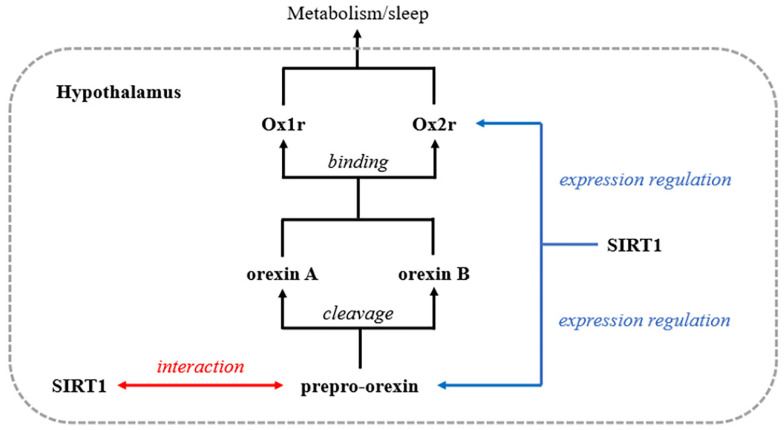
Relationship between SIRT1 and orexins in the normal physiological and pathological environment of ALS (SOD1G93A mice). SIRT1: Silent mating type information regulation 2 homolog 1; Ox1r: Orexin type 1 receptor; Ox2r: Ox1r: Orexin type 2 receptor; Black line: the way of orexins produces biological functions; Blue line: association between orexins and SIRT1 under normal physiological environment; Red line: additional association between orexins and SIRT1 in the ALS pathological environment (SOD1G93A mice).

Prepro-orexin is produced by orexinergic neurons in the lateral hypothalamus and is divided into orexin A and orexin B. Orexins A and B activate monoaminergic and cholinergic neurons in the hypothalamus and brain stem to maintain a long, consolidated awake period [12]. In addition, orexins promote food intake while supporting energy expenditure based on an organism’s nutritional and metabolic states to maintain energy balance [13]. SIRT1 can promote the orexin system by influencing the synthesis of orexins and their receptors. In aged mice, SIRT1-mediated regulation of energy expenditure and sleep quality is partly mediated by adjustment of the expression of the orexin type 2 receptor in the hypothalamus [14,15]. Panossian et al. found that SIRT1 mRNA present in orexinergic neurons shows a distinctive nuclear localization, while prepro-orexin mRNA levels are reduced in the hypothalamus in SIRT1-null C57BL/6J mice [16]. However, all these findings have been obtained under non-neurodegenerative conditions, and both external and internal environments are altered in ALS. Whether there is further contact between orexins and SIRT1 in the ALS pathological state remains unknown. Our study demonstrates that in addition to regulating the expression of prepro-orexin and its receptor, SIRT1 interacts with prepro-orexin directly in the hypothalamus in SOD1G93A transgenic mice (Figure 4). This finding provides new evidence of the relationship between these two molecules under ALS conditions. Increases in SIRT1 have a positive effect on ALS. Resveratrol, an activator of SIRT1, is beneficial for the viability of SOD1G93A cells in the pathological environment of ALS [17,18]. Animal studies have also shown that overexpression of SIRT1 delays motor symptom onset, improves motor function and improves survival [19,20]. Although our studies were unable to identify the effect of this interaction, we believe that the increase in SIRT1 in the hypothalamus is a kind of feedback mechanism protecting against the pathological changes of ALS.

Furthermore, we observed that the interaction appears even before motor symptom onset, when SIRT1 levels are not enhanced. Our previous study found that prepro-orexin is already expressed at high levels in the hypothalami of SOD1 mice at this time point even though SIRT1 levels are not elevated [9]. Therefore, we believe that this interaction is related only to the expression of orexin, not to that of SIRT1. This finding indicates that an increase in SIRT1 may be not only a protective response to neurodegeneration during ALS progression but also a response to overexpression of prepro-orexin. In other words, excessively elevated levels of prepro-orexin may deplete SIRT1 by interacting with it and diminish the protective effects of many pathways during disease progression. This mechanism may also explain why diet influences the onset and progression of disease in SOD1G93A mice, as we found in an earlier study (the decrease in orexin induced by a high-energy diet suppressed SIRT1 inhibition) [21]. Furthermore, this phenomenon can be explained by the possibility that the interaction is a negative feedback signal that directly reduces prepro-orexin mRNA synthesis by inhibiting SIRT1.

There are some limitations of our study. First, orexin A and orexin B levels are also increased in the hypothalamus in SOD1G93A mice, and there may be interactions between SIRT1 and these two proteins; however, we did not test these interactions. Second, we mentioned the interactions and hypothesized that prepro-orexin may diminish the effect of SIRT1 in ALS, but how the activity of SIRT1 changes if the interaction changes is unclear. We will address these limitations in the future to provide further evidence of the relationships between SIRT1 and orexins.

## 5. Conclusions

We observed increased SIRT1 levels in symptomatic ALS SOD1G93A transgenic mice and found that SIRT1 not only is a crucial molecule for orexin synthesis but also directly interacts with increased levels of prepro-orexin in the hypothalamus in both presymptomatic and symptomatic SOD1G93A transgenic mice. Our findings confirm that SIRT1 plays an important role in the pathological changes in the hypothalamus in ALS even before the onset of motor changes, revealing SIRT1 as a potential target for therapeutic interventions to improve metabolic imbalance and sleep/wake disorder and even slow disease progression.
